# Maternal Immunity and Vaccination Influence Disease Severity in Progeny in a Novel Mast Cell-Deficient Mouse Model of Severe Dengue

**DOI:** 10.3390/v13050900

**Published:** 2021-05-12

**Authors:** Chinmay Kumar Mantri, Gayathri Soundarajan, Wilfried A. A. Saron, Abhay P. S. Rathore, Sylvie Alonso, Ashley L. St. John

**Affiliations:** 1 Program in Emerging Infectious Diseases, Duke–National University of Singapore Medical School, Singapore 169857, Singapore; gaya.rajan@gmail.com (G.S.); wilfried.saron@duke-nus.edu.sg (W.A.A.S.); 2 Department of Pathology, Duke University Medical Center, Durham, NC 27710, USA; abhay.rathore@duke.edu; 3 Infectious Diseases Translational Research Programme, Department of Microbiology and Immunology, Yong Loo Lin School of Medicine, National University of Singapore, Singapore 117545, Singapore; micas@nus.edu.sg; 4 Immunology Programme, Life Sciences Institute, National University of Singapore, Singapore 119077, Singapore; 5 SingHealth Duke-National University of Singapore Global Health Institute, Singapore 168753, Singapore

**Keywords:** dengue, maternal immunity, mast cells, vaccines

## Abstract

Sub-neutralizing concentrations of antibodies in dengue infected patients is a major risk factor for the development of dengue hemorrhagic fever and dengue shock syndrome. Here, we describe a mouse model with a deficiency in mast cells (MCs) in addition to a deficiency in Type-I and II IFN receptors for studying dengue virus (DENV) infection. We used this model to understand the influence of MCs in a maternal antibody-dependent model of severe dengue, where offspring born to DENV-immune mothers are challenged with a heterologous DENV serotype. Mice lacking both MCs and IFN receptors were found susceptible to primary DENV infection and showed morbidity and mortality. When these mice were immunized, pups born to DENV-immune mothers were found to be protected for a longer duration from a heterologous DENV challenge. In the absence of MCs and type-I interferon signaling, IFN-γ was found to protect pups born to naïve mothers but had the opposite effect on pups born to DENV-immune mothers. Our results highlight the complex interactions between MCs and IFN-signaling in influencing the role of maternal antibodies in DENV-induced disease severity.

## 1. Introduction

Dengue is a mosquito-borne Flaviviral disease endemic in many countries within the tropical and subtropical regions of the world. It can be caused by any one of the four serotypes of the dengue virus (DENV1-4) [1]. Infection with the virus is mostly self-limiting and quickly resolves within weeks, with or without mild symptoms, such as fever, rash, and myalgia. However, in some cases, DENV infection can manifest as a more severe form of disease with the possibilities of hemorrhaging and plasma leakage, which can lead to multiple organ failure. These severe forms of dengue disease have been referred to as dengue hemorrhagic fever and dengue shock syndrome (DHF/DSS) [2,3]. In addition to vascular and respiratory dysfunction, dengue infection can also rarely lead to neurological and neuromuscular complications such as Guillain–Barre syndrome and hypokalemic paralysis [4,5,6].

The innate immune response is key for early infection control of DENV, and this involves not only innate immune responses in cells that are infected with DENV, such as monocytes and dendritic cells, but also a pro-inflammatory cellular immune response in the skin involving activation of mast cells (MCs), which are key for recruiting and activating cytotoxic cells including NK cells and various subsets of T cells including γδT cells [7,8]. MCs are abundant in the skin where the virus is injected by the mosquito, and they have been shown to influence the disease outcomes in multiple mouse models [9,10]. In contrast to evidence that MCs can contribute to severe vascular leakage at the systemic stage of disease [11], local infection in the skin is constrained by MCs, thus MC-deficient mice display heightened viral titers in the skin and draining lymph nodes, which are the secondary site of infection [7].

At a molecular level, interferons (IFNs), which are key for constraining DENV immune responses, are produced by multiple cell types, including those that are infected with DENV and as well as other bystander immune cells. AG129 mice, which are deficient in type-I and type-II IFNs, have been widely used as a model for infections, including DENV [12]. AG129 mice are particularly susceptible to DENV infection if the mice are sensitized with DENV2-specific but flavivirus cross-reactive antibody, 4G2, prior to DENV infection [13,14]. Both type-I and type-II IFNs induce an antiviral state that is mediated by both innate and adaptive immune cells. Type-I IFNs such as IFN-α and IFN- β are produced in response to viral infection and exert their effects through binding to IFN-α/β receptors (Ifnar). Although DENV induces a strong IFN-α/β response both in humans and mice, it can evade the IFN response by various mechanisms [15], including ones mediated through its non-structural (NS) proteins [16,17]. Type-I IFNs induce the expression of MHC-I molecules on virus-infected cells, increase the killing potential of natural killer (NK) cells, and stimulate Th1 cells. IFN-α has been shown to induce B cell proliferation and development of plasma cells and to induce IgG2a levels, and reduce IgG1 and IgE levels [18]. Type-II IFN, IFN-γ, being the central effector of cell-mediated immunity, coordinates many components of the antiviral response, such as increased antigen presentation, increased reactive oxygen species production, nitric oxide production, and others, simultaneously inhibiting all stages of the virus replication [19]. IFN-γ, a Th1 cytokine produced by Th1 cells, such as NK cells and NKT cells, is also known to fine-tune the humoral immune response by controlling the immunoglobulin isotypes produced by B cells [20]. IFN-γ has been reported to specifically inhibit IgG1, a major component of total IgG, with no notable effects on either IgG3 or IgG4 production levels [21]. LPS-activated murine B cells could be induced to produce increased levels of IgG2a upon treatment with IFN-γ [22]. However, intriguingly, it was also recently shown that early but transient IFN blockade could improve antibodies raised to viral vaccines, potentially by facilitating their early replication in vivo, allowing for a more robust early adaptive response [23]. In spite of this contrasting information indicating that the role of IFNs in infection control and immune activation can be complex, the increased virus replication and heightened severity of DENV disease in IFN-deficient mouse models are likely attributable to the role of IFNs in the early containment of viral infection.

Infection with one serotype of DENV usually confers life-long protection against the same serotype, but serotypic immunity can also be detrimental and put an individual at a greater risk of developing the severe form of the disease from a subsequent heterotypic DENV infection [24]. This has been explained by a mechanism called antibody-dependent enhancement (ADE) of disease, and it is thought to explain why pre-existing heterologous immunity is one of the most important risk factors for the development of severe dengue, along with age, sex, pre-existing metabolic diseases, asthma, and a genetic predisposition [25,26]. While it has also been shown that pre-existing heterotypic antibodies can augment the activation of immune cells leading to heightened inflammation [27,28], ADE has been attributed to the waning concentration of antibodies which are weakly or non-neutralizing to the secondary heterotypic infecting DENV, leading to enhanced uptake of virus particles and increased intracellular viral replication [29]. Associated largely with antibodies in an intermediate concentration range, ADE has been proposed based on epidemiological data [29]. This phenomenon can be observed in infants born to dengue-immune mothers who have been shown to be most at risk of developing severe dengue between 4–12 months of age, as maternal dengue antibodies wane [30]. Recently, this phenomenon of ADE and increased susceptibility to disease owing to maternal antibodies have been demonstrated in a mouse model, where young mice born to DENV-immune mothers were found to be susceptible to enhanced disease and infection upon heterologous DENV challenge [31,32]. Subsequent studies showed that most of these antibodies were obtained via breast milk [33]. Apart from ADE, pre-existing immunity has also been suggested to reactivate low affinity T cells during heterotypic DENV infection [34], although these are not transferred from mother to fetus, in contrast to antibodies that have an active transport mechanism across the placenta, [35], and that can also be acquired during breastfeeding [33]. Therefore, the heightened risk of severe dengue in infants specifically, is thought to be mainly driven by maternal antibodies. These studies also emphasize the importance of understanding the role that maternal antibody quality, in terms of titer, virus-directed binding sites, or avidity, has on the disease outcomes in progeny as aims to increase dengue vaccine coverage and efficacy could influence the maternal antibody-dependent responses.

Given the protective roles of IFNs and MCs, we generated mice lacking MCs and type-I and/or type-II IFN receptors to determine if DENV infection in these mice recapitulated signs of dengue and/or produced a more severe disease compared to the parent strains. Surprisingly, we found that prior DENV infection or vaccination of female mice had a differential influence on the severity of heterologous DENV infection in offspring, depending on the presence of Type-I or Type-II IFN in the MC-deficient model. Our findings thus indicated that the IFN system regulates the influence of maternal antibodies in progeny on disease severity.

## 2. Materials and Methods

### 2.1. Animal Studies

All animal experiments were conducted in the vivarium at Duke-NUS Medical School and according to protocols approved by the SingHealth Institutional Animal Care and Use Committee (2015/SHS/1048, 22 June 2015). MC-deficient Sash mice (*Kit^W-sh/W-sh^*) were originally purchased from The Jackson Laboratory and bred in-house. AG129 breeders were purchased from B&K Universal and bred at InVivos, Singapore. AG129 mice were crossed with Sash mice, and the progeny of these mice were then crossed with each other. F2 mice were segregated based on coat color, and *Ifnar1* deficiency was determined by PCR using primers 25375: ACT CAG GTT CGC TCC ATC AG, 25376: CTT TTA ACC ACT TCG CCT CGT, 27842: GAA CCT GAG GCT GTC GAA GG. Type-I deficient mice (*Ifnar1^−/−^*) gave a band of 450 bp, wild type mice (*Ifnar1^+/+^*) produced a band of 395 bp, and heterozygotic mice (*Ifnar1^+/−^*) produced both 395 bp, 450 bp bands. Similarly, *Ifngr1* deficiency was determined by PCR using primers 10774: CTC GTG CTT TAC GGT ATC GC, 22360: TCG CTT TCC AGC TGA TGT ACT, and 22361: CCA CCT CAG CAC TGT CTT CA. Type-II deficient mice *(Ifngr1^−/−^*) gave a band of 200 bp, wild type allele *(Ifngr1^+/+^*) produced a band of 429 bp, and heterozygotic *(Ifngr1^+/−^*) mice produced both 200 bp, 429 bp bands. After initial crossing, the desired genotypes on a mixed background were bred in-house to maintain the colony of each genotype. Mice from the F2 generation on the same mixed background, having the *Kit^+/+^Ifnar1^+/+^Ifngr1^+/+^* genotype, were used as wild-type mice, and similarly, *Kit^W-sh/W-sh^ Ifnar1^+/+^Ifngr1^+/+^* were used as MC-deficient mouse controls. Mice were reconstituted with 1 × 10^7^ mature bone marrow-derived mast cells (BMMCs) from congenic controls by intravenous injection. BMMCs were produced according to published protocols [7].

### 2.2. Virus Production

DENV1-4 (EDEN strains) [36] and D2Y98P [31] were produced in C6/36 mosquito cells in complete RPMI 1640 supplemented with 25 mM HEPES and 2% fetal bovine serum (FBS) at 28 °C and harvested 5 days after infection. Viral titers were determined by a standard plaque assay using BHK-21 cells [7,37].

### 2.3. Infections and Immunizations

Mice (6–10 weeks old) were infected with 1 × 10^6^ PFU DENV1-4 in 100 µL by intraperitoneal (i.p). injection (*n* = 8–9 per group) or 1 × 10^3^ PFU of D2Y98P subcutaneously (s.c) (*n* = 5–10 per group). For immunization, 1 × 10^5^ PFU of DENV1 were s.c. injected after 30 min ultraviolet light inactivation. The clinical symptoms were evaluated and scored as follows: 0—at healthy state, 1—signs of ruffled fur, 2—hunched back, 3—severe diarrhea, 4—moribund stage, 5—severe weight loss. Temperature was monitored daily using a rectal probe. For survival curves, mice were monitored daily for humane endpoints.

### 2.4. Serum IgG Endpoint Titer

Black 96-well half-area microtiter plates (Costar, Corning, USA) were coated with 1 × 10^5^ PFU/mL of DENV1 (Eden strain) in carbonate buffer (15 mM Na_2_CO_3_, 35 mM NaHCO_3_, pH 9.6) overnight at 4 °C. Plates were blocked with 3% non-fat dry milk powder in PBS with 0.05% Tween-20, for 2 h at room temperature. Plates were washed and 2-fold serial dilutions of mouse sera in complete sample diluent (PBS, BSA 1%, non-fat dry milk 1% W/V, normal goat serum 5% *v*/*v* (Sigma-Aldrich, St. Louis, USA ), and Tween-20 0.05% *v*/*v*), beginning at 1:16, were added and incubated overnight at 4 °C. After washing, alkaline phosphatase–conjugated anti-mouse IgG detection antibody (SouthernBiotech, Birmingham, USA) was added at 1/8000 dilution in secondary antibody diluent (PBS, BSA 0.5%, normal goat serum 5% (Sigma-Aldrich, St. Louis, USA), and 0.05% Tween-20 and incubated for 2 h at room temperature. After washing, plates were incubated with Attophos AP fluorescent substrate system (Promega, Madison, USA) for 45 min at room temperature in the dark and then read at 440 nm (excitation)/560 nm (emission) using a plate reader (Spark 10 M, Tecan, Männedorf, Switzerland). Serum dilutions were considered positive if the relative light unit (RLU) measured was at least two-fold higher than the value of the naïve serum at the same dilution.

### 2.5. DENV Genome Quantification in Serum

RNA was isolated from pooled serum samples using the RNeasy Kit (Qiagen, Hilden, Germany) according to the manufacturer’s protocol. Quantitative real-time PCR was achieved with SuperScript™ III Platinum™ One-Step qRT-PCR Kit (Invitrogen, Carlsbad, USA) using primer pair C14A (AAT ATG CTG AAA CGC GAG AGA AAC CGC G) and C69B (CCC ATC TCI TCA IIA TCC CTG CTG TTG G) with probe VICD2C388 (AGC ATT CCA AGT GAG AAT CTC TTT GTC AGC TGT) in a CFX96 Touch Real-Time PCR Detection System (Bio-Rad, Hercules, USA) [38]. Copy numbers were then calculated using standard curves generated from serial dilutions of DNA plasmids containing DENV genome sequence.

### 2.6. Statistical Analysis

Statistical significance was determined by one-way or two-way ANOVA using GraphPad Prism (GraphPad Software, San Diego, USA). A *p* value <0.05 was considered significant. For all graphs, error bars represent the SEM.

## 3. Results

### 3.1. Mice Lacking MCs in Addition to Type-I and Type-II Interferon Receptors Are Susceptible to DENV Infection

To investigate how MCs influence maternal antibody-mediated dengue disease, we first generated mice that lacked both MCs and IFN receptors by crossing AG129 (*Ifnar1^−/−^ Ifngr1^−/−^*) mice with MC-deficient Sash (*Kit^W-sh/W-sh^*), and the F1 progeny were then crossed with each other. F2 progeny mice were then segregated based on their *c-kit* genotype and deficiency in both IFN receptors. The resulting mice included several key desired genotypes: Mice lacking both MCs and IFN receptors (*Kit^W-sh/W-sh^ Ifnar1^−/−^ Ifngr1^−/−^*), mice lacking MCs and type-I IFN receptor (*Kit^W-sh/W-sh^ Ifnar1^−/−^ Ifngr1^+/+^*), and mice lacking MCs and type-II IFN receptor (*Kit^W-sh/W-sh^ Ifnar1^+/+^Ifngr1^−/−^*).

**Figure 1:**
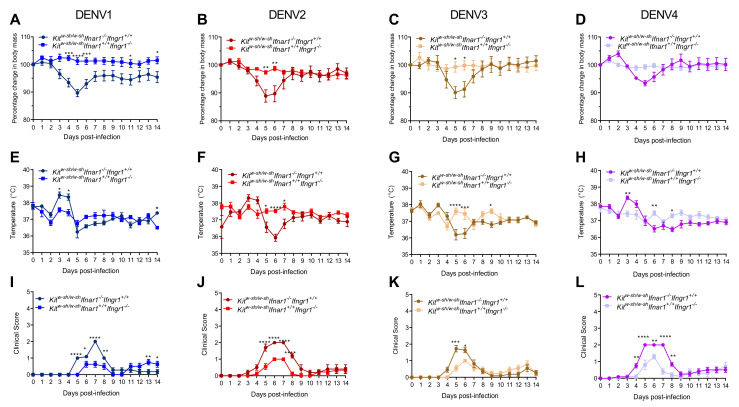
Type-I IFN deficiency worsens dengue disease severity compared to type-II IFN deficiency in MC-deficient mice. *Kit^W-sh/W-sh^ Ifnar1^−/−^ Ifngr1^+/+^* and *Kit^W-sh/W-sh^ Ifnar1^+/+^ Ifngr1^−/−^* mice were infected with 1 × 10^6^ PFU of DENV1–4 (EDEN strain) by i.p. injection. Change in (**A**–**D**) body mass, (**E**–**H**) body temperature, and (**I**–**L**) clinical scores were recorded every day for two weeks. Data represent mean ± SEM, * *p* < 0.05, ** *p* < 0.01, *** *p* < 0.001, and **** *p* < 0.0001, by two-way ANOVA with Holm-Sidak’s post-test. For all panels, *n* = 8–9 mice per group.

To characterize DENV infection in mice lacking MCs and type-I and/or type-II IFN receptors, adult mice were i.p. infected with 1 × 10^6^ PFU of DENV1, DENV2, DENV3, or DENV4 and were monitored for 14 days. This dose was selected as it was shown to be non-lethal in AG129 mice without antibody-dependent enhancement [39]. We first compared the clinical manifestations displayed by MC-deficient *Kit^W-sh/W-sh^ Ifnar1^+/+^ Ifngr1^−/−^* mice lacking Type-II IFN receptors and MC-deficient *Kit^W-sh/W-sh^ Ifnar1^−/−^ Ifngr1^+/+^* mice lacking Type-I IFN receptors. We observed that in spite of displaying signs of disease, all mice survived infection with DENV1-4 in these models. MC-deficient mice lacking type-I IFN receptor lost weight from day 3 post-infection when infected with each of the four DENV serotypes and gradually recovered after day 5, while MC-deficient mice lacking type-II IFN receptor did not experience any significant weight loss, although they also did not gain weight during the 2-week study duration (Figure 1A–D). A drop in body temperature was observed from day 3 onwards for the mice lacking type-1 IFN signaling during infection with each of the four DENV serotypes, while mice lacking type-II IFN receptors did not display significant changes in body temperature (Figure 1E–H). Coinciding with the weight loss and body temperature changes, MC-deficient mice lacking type-I IFN receptors showed a higher clinical score compared to MC-deficient mice lacking type-II IFN receptors, and the disease symptoms started around day 4 and mostly resolved by day 9 for each DENV serotype (Figure 1I–L). Given the indications that MC-deficient mice lacking either type-I or type-II IFN receptors display morbidity during infection by DENV1-4, we proceeded to investigate whether double-knockouts lacking MCs as well as both type-I and type-II IFN receptors showed more severe disease.

**Figure 2:**
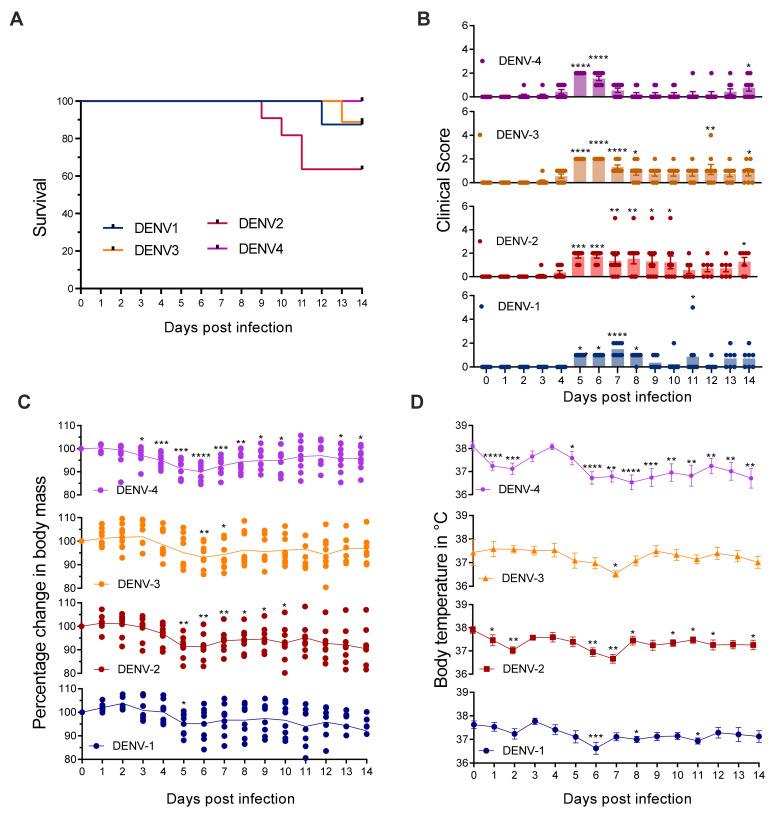
*Kit^W-sh/W-sh^ Ifnar1^−/−^ Ifngr1^−/−^* mice are susceptible to DENV infection. Mice (*n* = 8–11) were infected i.p. with 1 x 10^6^ PFU of DENV1, DENV2, DENV3, or DENV4 (EDEN strains). (**A**) Survival curve for adult *Kit^W-sh/W-sh^ Ifnar1^−/−^ Ifngr1^−/−^* (**B**) Clinical score of DENV infected mice 1: ruffled fur, 2: hunch back, 3: diarrhea, 4: paralysis and moribund 5: death. (**C**) Change in body mass, Line represents the average, and dots represent individual mouse and (**D**) temperature following DENV infection. Data represent mean ± SEM, * *p* < 0.05, ** *p* < 0.01, *** *p* < 0.001, and **** *p* < 0.0001, by one-way ANOVA with Dunnett’s post-test.

Infection of MC-deficient *Kit^W-sh/W-sh^ Ifnar1^−/−^ Ifngr1^−/−^* mice lacking both type-I and type-II IFN receptors with DENV1-4 caused varying degrees of mortality and morbidity. DENV2 infection was most severe and resulted in 40% mortality (Figure 2A). Infection with DENV1 or DENV3 resulted in death for 10% of mice, while all DENV4-infected mice survived (Figure 2A). All of the DENV serotypes caused morbidity in *Kit^W-sh/W-sh^ Ifnar1^−/−^ Ifngr1^−/−^* mice, as indicated by the significantly increased clinical score at various days between day 2 to day 8 post-infection (Figure 2B). Consistent with the survival curve, mice infected with DENV2 showed the highest clinical score (Figure 2B), and 2 out of 10 mice showed signs of paralysis in hind limbs when humane endpoints were met. Clinical scores were similar between DENV1 and DENV4, while mice infected with DENV3 showed significant morbidity by day 5, and their clinical scores remained high until day 8 (Figure 2B). There was also a significant drop in body mass (Figure 2C) and temperature (Figure 2D) of *Kit^W-sh/W-sh^ Ifnar1^−/−^ Ifngr1^−/−^* upon infection with DENV. Since *Kit^W-sh/W-sh^ Ifnar1^−/−^ Ifngr1^+/+^* and *Kit^W-sh/W-sh^ Ifnar1^−/−^ Ifngr1^−/−^* mice were infected with DENV2 in the subsequent experiments, we also quantified the viremia on day 3 post-infection for this group, during acute infection, and on day 10 post-infection to check for viral clearance. DENV2 could be detected in the serum of both *Kit^W-sh/W-sh^ Ifnar1^−/−^ Ifngr1^+/+^* and *Kit^W-sh/W-sh^ Ifnar1^−/−^ Ifngr1^−/−^* mice during acute infection (Supplementary Figure S1). Mice with a functional IFN-γ response cleared the infection completely by day 10, even in the absence of MCs and type-1 IFN. Viral load was much lower in *Kit^W-sh/W-sh^ Ifnar1^−/−^ Ifngr1^−/−^* mice on day 10 post-infection than day 3, indicating clearance of the virus (Supplementary Figure S1). These results indicate that mice lacking both MCs and IFN could be a valuable tool for studying DENV, allowing more severe disease outcomes than respective controls and morbidity and mortality at moderate DENV doses during primary infection.

### 3.2. Progeny of DENV1 Immune Kit^W-sh/W-sh^ Ifnar1^−/−^ Ifngr1^−/−^ Mothers Are Protected from Subsequent Heterologous DENV2 Infection

**Figure 3:**
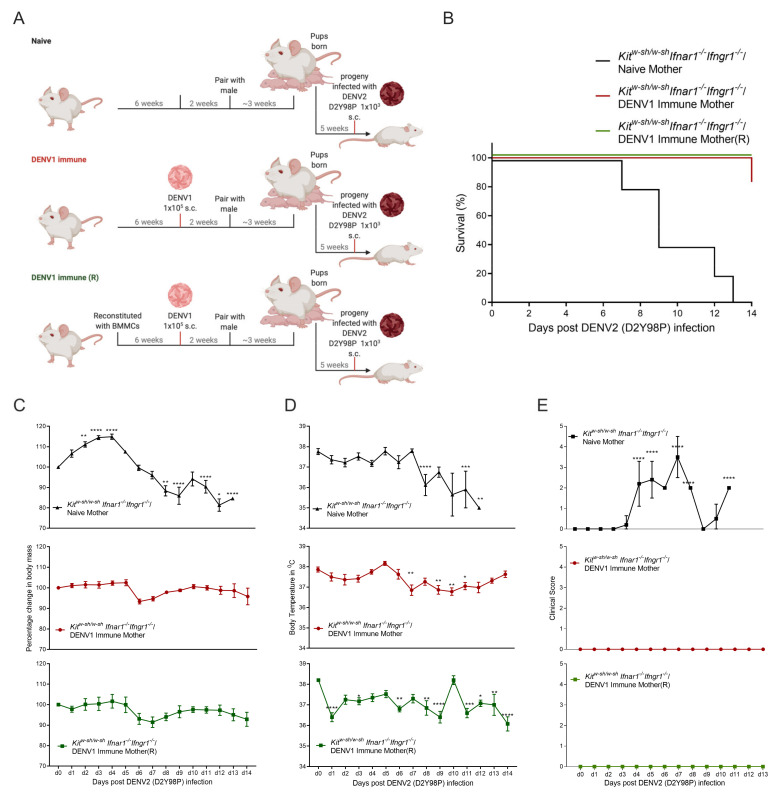
Maternal DENV antibody provides protection to pups in *Kit^W-sh/W-sh^ Ifnar1^−/−^ Ifngr1^−/−^* mice. (**A**) Experimental timeline and depicting maternal exposure to DENV1 and/or MC-reconstitution for naïve or DENV1- immune mice followed by mating and exposure of progeny to DENV2. (**B**) Survival curve of pups born to naïve (*Kit^W-sh/W-sh^ Ifnar1^−/−^ Ifngr1^−/−^/*Naïve Mother) or DENV1-immune mice, with (*Kit^W-sh/W-sh^ Ifnar1^−/−^ Ifngr1^−/−^/*DENV1 Immune Mother(R)) and without MC reconstitution (*Kit^W-sh/W-sh^ Ifnar1^−/−^ Ifngr1^−/−^/*DENV1 Immune Mother) after DENV2 (D2Y98P) infection, as diagramed in panel A (*n* = 5–6). (**C**) Change in body mass and (**D**) temperature and (**E**) clinical score following DENV2 (D2Y98P) infection. Data represent mean ± SEM, * *p* < 0.05, ** *p* < 0.01, *** *p* < 0.001, and **** *p* < 0.0001, by one-way ANOVA with Dunnett’s post-test.

ADE of DENV has been demonstrated in AG129 mice lacking type-I and type-II IFN receptors and it has been shown that pups born to DENV-immune mothers are more susceptible to heterologous DENV challenge [40]. AG129 mice born to DENV-immune dams have been demonstrated to be more vulnerable to subsequent heterologous DENV challenges in an ADE-dependent manner at 5 weeks post-birth [31]. Although MCs have been shown to promote antibody responses during vaccination [41], the role of MCs in antibody-mediated functions in the context of viral infection is unknown. To study how MCs influence maternal antibody-mediated outcomes during DENV infection, we utilized the previously described mouse model in which maternal antibodies have been shown to protect the pups up to 3 weeks after birth, but to cause ADE after that [31]. To study the maternal antibody-mediated response in progeny in the absence of MCs, *Kit^W-sh/W-sh^ Ifnar1^−/−^ Ifngr1^−/−^* adult female mice were first infected with DENV1, as shown in Figure 3A, and 2 weeks post-infection, after the virus has been cleared, were paired with DENV-naïve male counterparts. To confirm the presence of maternally-acquired antibody, serum from pups born to naïve- and DENV1-infected dams were used to detect DENV1-specific IgG at week 5 post-birth (Supplementary Figure S2). Pups born to naïve dams or DENV1-infected dams were then infected with 1 × 10^3^ DENV2 (using the highly virulent D2Y98P strain) at 5 weeks of age (Figure 3A). As expected, mice born to naïve mothers were susceptible to cutaneous DENV2 challenge with this highly virulent strain, and 90% of the pups died between day 7 to day 13, with one mouse showing paralysis of hind limbs when humane endpoints were met (Figure 3B). Although these mice initially gained weight for 4 days and appeared normal, they started showing morbidity from day 5 and there was a rapid decrease in body mass and temperature (Figure 3C). To control for the possibility of the mother’s MCs influencing the antibody responses, we also included a group of *Kit^W-sh/W-sh^ Ifnar1^−/−^ Ifngr1^−/−^* mice that were first reconstituted with IFN-sufficient bone marrow-derived mast cells (BMMCs) from WT mice (*Kit^W-sh/W-sh(**R**)^ Ifnar1^−/−^ Ifngr1^−/−^*) prior to infection with DENV1 (as diagramed in Figure 3A). The progeny of both MC-deficient and MC-reconstituted DENV-immune mice were protected from heterologous DENV2(D2Y98P) challenge (Figure 2B). We also compared the change in body mass (Figure 3C) and temperature (Figure 3D) of the DENV2-infected mice born to *Kit^W-sh/W-sh^ Ifnar1^−/−^ Ifngr1^−/−^* mice to the DENV2-infected progeny of DENV1-immune *Kit^W-sh/W-sh^ Ifnar1^−/−^ Ifngr1^−/−^* mice, with and without MC-reconstitution. The DENV1-immune groups did not show significantly decreased body mass, and the drop in temperature they experienced was less severe (Figure 3C,D). These two DENV1-immune groups also showed a less severe clinical scores compared to the naïve group and their body mass recovered from day 7 onward (Figure 3E), supporting that the maternal antibody response was still protective at 5 weeks after birth in this model, even in the absence of MCs, which is longer than was observed in the AG129 model [31].

### 3.3. Increased Disease Severity of Heterologous DENV2 Infection in Progeny of DENV1-Immune Kit^W-sh/W-sh^ Ifnar1^−/−^ Ifngr1^+/+^ Mice

**Figure 4:**
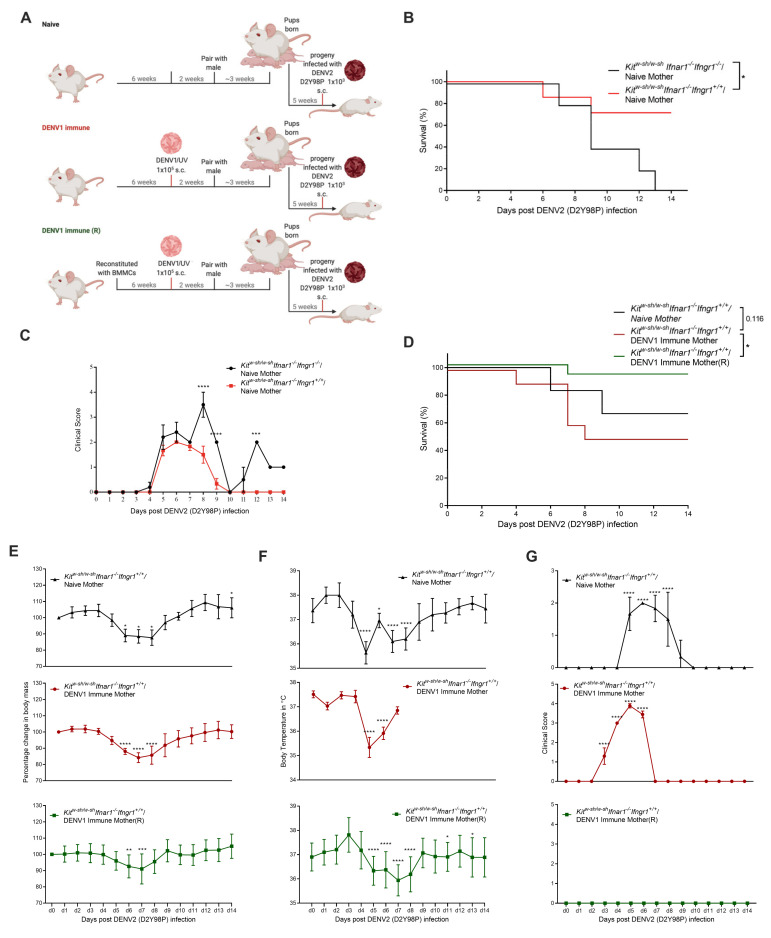
Maternal antibody produced in the presence of MCs provides protection to DENV-infected *Kit^W-sh/W-sh^ Ifnar1^−/−^ Ifngr1*^+/+^ progeny. (**A**) Experimental timeline depicting maternal vaccination against DENV1 and/or MC-reconstitution for naïve or DENV1-immune mice, followed by mating and exposure of progeny to DENV2. (**B**) Data reproduced from Figure 3B,D in a new graph for statistical comparison of survival of DENV2 infected progeny of DENV-naïve *Kit^W-sh/W-sh^ Ifnar1^−/−^ Ifngr1*^−/−^ versus *Kit^W-sh/W-sh^ Ifnar1^−/−^ Ifngr1*^+/+^ mice. (**C**) Data reproduced from Figure 3E and Figure 4G in a new graph for statistical comparison of the clinical score of DENV2 infected progeny of DENV-naïve *Kit^W-sh/W-sh^ Ifnar1^−/−^ Ifngr1*^−/−^ versus *Kit^W-sh/W-sh^ Ifnar1^−/−^ Ifngr1*^+/+^ mice. (**D**) Survival curve of the progeny of naïve and DENV-immune dams with genotypes corresponding to the presence (*Kit^W-sh/W-sh(R)^ Ifnar1^−/−^ Ifngr1*^+/+^) or absence (*Kit^W-sh/W-sh^ Ifnar1^−/−^ Ifngr1*^+/+^) of MCs in the mothers (*n* = 6–12). Change in (**E**) body weight (**F**) temperature and (**G**) clinical score following DENV2 infection of the mouse groups displayed in panel A. Data represent mean ± SEM, * *p* < 0.05, ** *p* < 0.01, *** *p* < 0.001, and **** *p* < 0.0001, by one-way ANOVA with Dunnett’s post-test.

We next investigated the role of maternal DENV immunity on heterologous DENV infection in mice lacking Type-I but not Type-II IFN receptor and MCs (*Kit^W-sh/W-sh^ Ifnar1^−/−^ Ifngr1^+/+^*). Surprisingly, we found that *Kit^W-sh/W-sh^ Ifnar1^−/−^ Ifngr1^+/+^* mice born to naïve mothers were particularly vulnerable to s.c. DENV1 challenge, and all the infected mice died by day 8 post-infection in contrast to infection by i.p. injection where all the mice survived (Supplementary Figure S3), suggesting that the route of infection was critical to the influence of immunity and that skin-resident MCs are likely protective. Thus, to maintain the ability to challenge the progeny of these mice, we chose to immunize the mice through the s.c. route, using UV-inactivated DENV1 virus, before pairing with the males of the same genotype (Figure 4A). Again, a group of MC-reconstituted dams was also included to control for the possible influence of maternal MCs on antibody production (Figure 4A). Infection of the progeny mice born to naïve *Kit^W-sh/W-sh^ Ifnar1^−/−^ Ifngr1^+/+^* mothers, with DENV2(D2Y98P) s.c., resulted in 30% mortality, compared to 90% mortality in *Kit^W-sh/W-sh^ Ifnar1^−/−^ Ifngr1^−/−^* mice (Figure 4B) and lower clinical score (Figure 4C), indicating the protective property of IFN-γ signaling. Mice born to DENV1 immune *Kit^W-sh/W-sh^ Ifnar1^−/−^ Ifngr1^+/+^* dams were also more susceptible to subcutaneous DENV2 challenge and showed 50% mortality, suggesting enhancement of the disease (Figure 4D) similar to the previously established AG129 mice model [39]. This points to a critical role of IFN-α deficiency in the presence of IFN-γ in leading to enhancement of disease.

To study how MCs influence the maternal antibody-dependent outcomes, *Kit^W-sh/W-sh^ Ifnar1^−/−^ Ifngr1^+/+^*, we had also included a group where female mice were reconstituted with WT BMMCs prior to DENV1 immunization and pregnancy (Figure 4A). Indeed, the protective contributions of maternal MCs in this model could be observed since mice born to MC-reconstituted DENV1-immune mothers were protected from DENV2 heterologous challenge (Figure 4D) 5 weeks post-birth. The change in body mass (Figure 4E) and temperature (Figure 4F) were similar across groups, with all showing a drop in temperature or mass within the range of 4–8 days post-infection, and the surviving mice in each group showed similar recovery kinetics (Figure 4E,F). Interestingly, although the clinical score was elevated in the progeny of both naïve and DENV1-immune *Kit^W-sh/W-sh^ Ifnar1^−/−^ Ifngr1^+/+^* mothers, maternal MC-reconstitution had an effect of reducing the clinical score (Figure 4G). These results highlight the likelihood that MCs improve the antibody response such that maternal antibody maintains its protective capacity even in the context of Type-I IFN deficiency.

## 4. Discussion

The enhanced risk of severe dengue in infants in DENV-endemic settings is likely attributable to the presence of heterologous DENV-specific maternal antibodies [30]. It is thought that although initially, the neutralizing antibody titer is high enough to protect infants from DENV infection, the decay of maternal antibodies results in sub-neutralizing antibody concentrations that can cause ADE upon heterotypic DENV infection [30]. In support of this, in AG129 mice lacking type-I and type-II IFNs, progeny of DENV-immune mothers are protected up to an age of 2 weeks and 3 weeks, after which an increased disease severity was observed [31]. Here, we aimed to study the contributions of MCs to the maternal antibody-mediated enhancement of dengue disease since MCs are thought to influence antibody quality in terms of titer and avidity [42,43,44] and because they are critical immune sentinels of DENV. Indeed, animals lacking either Type-I or combined Type-I and -II IFNs that were MC-deficient were more susceptible to primary DENV infection and displayed morbidity. Unexpectedly, however, mice born to DENV-immune mothers with this same immune-compromised genotype (*Kit^W-sh/W-sh^ Ifnar1^−/−^ Ifngr1^−/−^)* were found to be protected against a heterologous DENV2 challenge. This could be due to differences in antibody production in our mouse model with a mixed genetic background compared to the AG129 mice that were used in prior reports [25] or other factors relating to immune regulation in MC-deficient mice. We have seen that the early adaptive immune response and virus clearance is slower in Sash mice compared to WT mice [8], which could potentially contribute to longer antigen persistence and influence antibody production. These observations, combined with our data demonstrating an influence of MCs on maternal antibody-mediated disease, emphasize the need for further studies to identify the influence of MCs on antibody responses and the role of IFNs in this process.

In contrast to mice that lack both Type-I and Type-II IFNs in addition to lacking MCs, MC-deficient mice that lacked only Type-I IFN displayed a different disease profile in the context of maternal antibody-mediated heterologous DENV disease. Interestingly, the presence of IFN-γ in the absence of Type-I IFNs was found to increase mortality and disease severity at 5 weeks of age in MC-deficient mice born to DENV-immune mothers compared to combined Type-I and –II IFN-deficient MC-deficient animals. Inhibition of Type-I IFN during Zika virus infection in adult mice has been reported to induce a robust Zika virus-specific IgG response and subtype switching [45]. Interestingly, it has been shown that early and transient restriction of the immune response, such as through transient IFN-blockade, can potentially lead to improved long-term antibody responses in the context of viral vaccines, even though the complete loss of Type-I IFNs is detrimental to antibody responses [23]. Our data suggest that maternal antibodies generated in the presence of IFN-γ and absence of Type-I IFNs and MCs resulted in more severe outcomes in progeny upon heterologous challenge. However, antibodies generated in the presence of MCs in MC-reconstituted mothers were protective, as indicated by better survival of pups born to these mothers. It’s interesting to note that MCs probably did not influence the antibodies in the absence of both Type-I and -II IFNs since we observed a similar disease profile irrespective of whether the mother had MCs or not. However, in the presence of only Type-II IFNs, MC reconstitution led to improved outcomes in progeny, likely through influencing antibody responses and potentially through compensating for the lack of Type-I IFNs.

Our results also have implications for mouse model development for DENV. We observed that mice lacking MCs and Type-I and -II IFNs were particularly susceptible to primary infections with DENV1-3, showing a mortality range of 10–40%, and yielded a range of symptoms that includes loss in body weight, hypothermia, ruffled fur, and diarrhea, even at this moderate inoculating dose of virus. Although our mice did not display DENV4 infection-induced mortality, their clinical scores were comparable to other DENV serotypes. Thus, combined deficiency in MCs and Type-I and Type-II IFNs is a potential way to achieve more severe and lethal disease during primary infection. This system contrasts other mouse models, which require high inoculating titers of virus combined with antibody-enhancement to achieve lethal disease [46]. Although the immune-compromised state makes this system less appropriate for mechanistic studies into immunity and pathogenesis, this model may still be useful for specific applications such as antiviral drug testing, especially where a severe model of primary infection is needed. Of additional interest is the fact that some mice infected with DENV3 showed symptoms of paralysis and neurological complications, which also have been reported rarely in dengue severe cases, including by DENV3 [47]. Thus IFN/MC-deficient mice could be used to study neurological effects of DENV infection and could be particularly important in pharmacological studies to screen drugs for dengue mediated neurological complications. These mice also highlight the importance of MCs providing protection against neurological symptoms since they only occurred in the combined absence of IFN and MCs. Another interesting aspect of our mouse model is that both of our mouse *Kit^W-sh/W-sh^ Ifnar1^−/−^ Ifngr1^−/−^* and *Kit^W-sh/W-sh^ Ifnar1^−/−^ Ifngr1^+/+^* were particularly susceptible to infection by the s.c. infection route, which is the natural route of DENV infection. Naïve mice with IFN-γ also showed less mortality and disease severity, which underscores the protective property of IFN-γ. Since IFN-γ is an effector cytokine for CD8 T cells, these data may suggest an important role for cytotoxic T cells during primary infection.

Together these findings using a model of maternal antibody-mediated enhanced DENV disease highlight the modulation of IFNs on maternal antibodies and support the potential protective role of MCs in generating antibodies in the IFN-deficient system. This could have implications for understanding immune influences on maternal antibody-mediated DENV disease as well as implications for improving DENV vaccines for women in DENV-endemic settings.

## Data Availability

All data needed to evaluate the conclusions of the study are contained within the figures.

## Supplementary Materials

The following are available online at https://www.mdpi.com/article/10.3390/v13050900/s1, Figure S1: Productive replication and clearance of DENV2 in *Kit^W-sh/W-sh^ Ifnar1^−/−^ Ifngr1^+/+^* and *Kit^W-sh/W-sh^ Ifnar1^−/−^ Ifngr1^−/−^* mice, Figure S2: Maternally acquired DENV1 IgG could be detected in *Kit^W-sh/W-sh^ Ifnar^1−/−^ Ifngr1^−/−^* mice, Figure S3: Subcutaneous DENV1 infection in *Kit^W-sh/W-sh^ Ifnar1^−/−^ Ifngr1^+/+^* mice was lethal.
