# Supplementary material for: Maternal Immunity and Vaccination Influence Disease Severity in Progeny in a Novel Mast Cell-Deficient Mouse Model of Severe Dengue
Source: Viruses. 2021 May 12;13(5):900. doi: 10.3390/v13050900 (PMC8152039; doi:10.3390/v13050900)
Supplement: Supplementary file 1 [file viruses-13-00900-s001.zip › viruses-1163904-supplementary.pdf]

viruses

MDPI

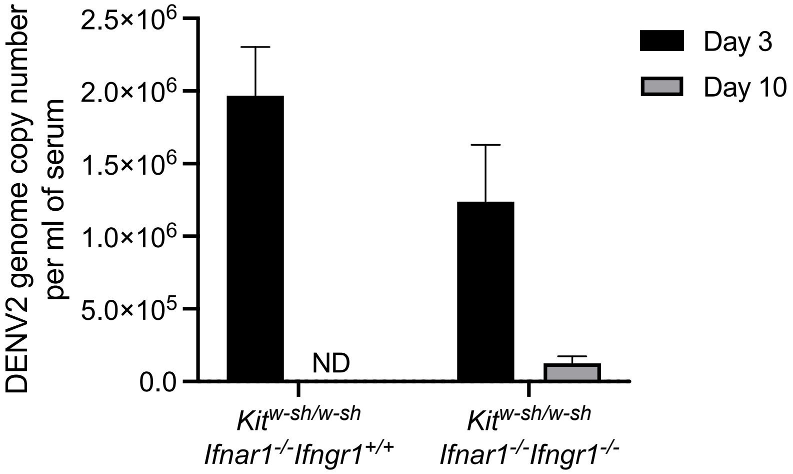

Supplementary Figure S1. Productive replication and clearance of DENV2 in *Kit*$^{W\text{-}sh/W\text{-}sh}$ *Ifnar1*$^{-/-}$ *Ifngr1*$^{+/+}$ and *Kit*$^{W\text{-}sh/W\text{-}sh}$ *Ifnar1*$^{-/-}$ *Ifngr1*$^{-/-}$ mice. 5–6 week-old mice (n = 5–10) were infected with 1 × 10^6 PFU of DENV2 (EDEN2 strain) by i.p. injection. Serum was collected on days 3 and day 10 post-infection and pooled before isolating viral RNA. Quantification of viral genome copies demonstrate productive replication in both *Kit*$^{W\text{-}sh/W\text{-}sh}$ *Ifnar1*$^{-/-}$ *Ifngr1*$^{+/+}$ and *Kit*$^{W\text{-}sh/W\text{-}sh}$ *Ifnar1*$^{-/-}$ *Ifngr1*$^{-/-}$ mice by day 3 post infection. While *Kit*$^{W\text{-}sh/W\text{-}sh}$ *Ifnar1*$^{-/-}$ *Ifngr1*$^{+/+}$ mice cleared infection completely by day 10, *Kit*$^{W\text{-}sh/W\text{-}sh}$ *Ifnar1*$^{-/-}$ *Ifngr1*$^{-/-}$ mice had very low level of infection by day 10. Data represent mean ± SEM, ND: Not detected.

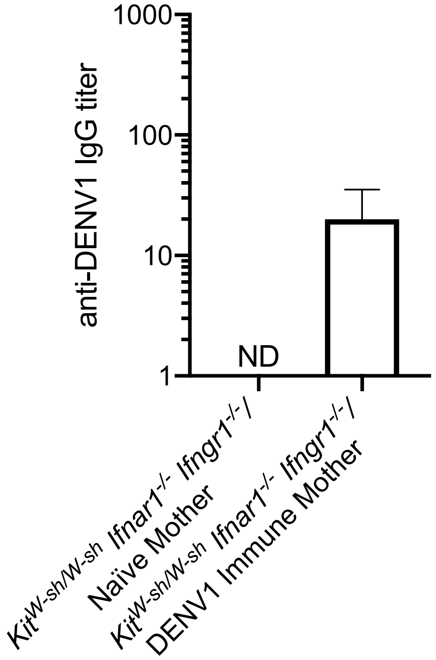

Supplementary Figure S2. Maternally acquired DENV1 IgG could be detected in *Kit*$^{W\text{-}sh/W\text{-}sh}$ *Ifnar1*$^{-/-}$ *Ifngr1*$^{-/-}$ mice. DENV1 specific IgG titre in serum collected from 5 weeks old *Kit*$^{W\text{-}sh/W\text{-}sh}$ *Ifnar1*$^{-/-}$ *Ifngr1*$^{-/-}$ mice born to naïve or DENV1 immunized mothers (n = 4–5). Data represent mean ± SEM,
ND: not detected.

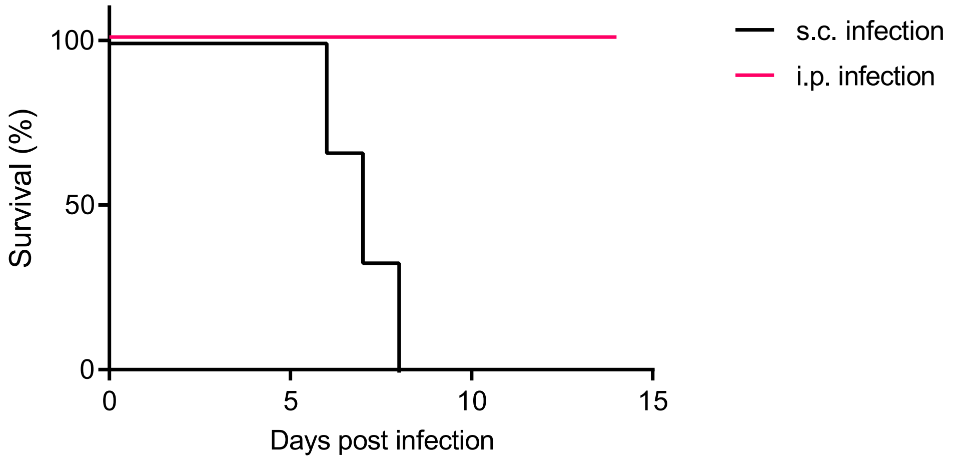

Supplementary Figure S3. **Subcutaneous DENV1 infection in *Kit*$^{\text{W-sh/W-sh}}$ *Ifnar1*$^{-/-}$ *Ifngr1*$^{+/+}$ mice was lethal**. 5-6 wk old female *Kit*$^{\text{W-sh/W-sh}}$ *Ifnar1*$^{-/-}$ *Ifngr1*$^{+/+}$ mice (n = 5) were infected with 1×10^5 PFU of DENV1 subcutaneously and monitored for 2wks for survival analysis.
